# Dynamic Risk Stratification Integrated with ATA Risk System for Predicting Long-Term Outcome in Papillary Thyroid Cancer

**DOI:** 10.3390/cancers15184656

**Published:** 2023-09-21

**Authors:** Laura Valerio, Cristina Dalmiglio, Fabio Maino, Elisa Mattii, Andrea Trimarchi, Alessandra Cartocci, Maria Grazia Castagna

**Affiliations:** 1 Department of Medical, Surgical and Neurological Sciences, University of Siena, 53100 Siena, Italy; laura.valerio@ao-siena.toscana.it (L.V.); cristina.dalmiglio2@unisi.it (C.D.); fabio.maino@ao-siena.toscana.it (F.M.); elisa.mattii1@gmail.com (E.M.); andrea.trimarchi@student.unisi.it (A.T.); 2 Department of Medical Biotechnologies, University of Siena, 53100 Siena, Italy; alessandra.cartocci@dbm.unisi.it

**Keywords:** postsurgical risk stratification, thyroid cancer, dynamic risk stratification, outcome

## Abstract

**Simple Summary:**

This study reports the clinical outcome of 704 papillary thyroid cancer (PTC) patients followed at our institution for a median time of 8 years. Currently, the long-term follow-up is based on the response to initial therapy (excellent response, indeterminate response, or biochemical or structural incomplete response) evaluated at 6–12 months after initial treatment. We assumed that the long-term follow-up of PTC patients might be better tailored by integrating the response to initial therapy with the American Thyroid Association (ATA) risk stratification system (low-, intermediate- and high-risk). This study confirmed that the initial response to therapy is predictive of the long-term outcome in PTC patients but also showed that the initial ATA risk class may be useful for improving the risk-adapted management of PTC patients based on the response to initial therapy.

**Abstract:**

Background: In recent years, there has been a renewed interest in thyroid cancer management paradigms that use individualized risk assessments as the basis for treatment and follow-up recommendations. In this study, we assumed that the long-term follow-up of differentiated thyroid cancer patients might be better tailored by integrating the response to initial therapy with the America Thyroid Association (ATA) risk classes. Methods: This retrospective study included low- and intermediate-risk papillary thyroid cancer (PTC) patients followed up for a median time of 8 years and classified according to the response to initial therapy assessed 6–12 months after initial treatment. Results: After a median follow-up of 8 years, in the initial excellent response subgroup of PTC patients (*n* = 522), the rate of recurrent disease was significantly higher in intermediate-risk patients than in low-risk PTC patients (6.9% versus 1.2%, *p* = 0.0005). Similarly, in the initial biochemical incomplete response subgroup (*n* = 82), the rate of excellent response was significantly higher in low-risk PTC patients (58.0%) than in intermediate-risk PTC patients (33.3%) (*p* = 0.007). Finally, in the initial structural incomplete response subgroup (*n* = 66), the rate of excellent response was higher in low-risk patients (80.0%) than in intermediate-risk patients (46.4%) (*p* = 0.08). Moreover, all patients with initial indeterminate response had an excellent response at the last follow-up visit. ATA risk classes were independently associated with long-term outcome in each subgroup of patients classified dynamically after initial therapy and the overall prognostic performance, defined via ROC curve analysis, of response to initial therapy integrated with the ATA risk system (AUC: 0.89; 95% CI: 0.86–0.92) was significantly higher compared to the ATA risk stratification (AUC 0.69; 95% CI: 0.65–0.74, *p* < 0.001) or the dynamic risk stratification (DRS) systems alone (AUC: 0.86 95% CI: 0.82–0.90, *p* = 0.007). Conclusions: This study of a large cohort of PTC patients showed that the initial ATA risk criteria may be useful for improving the risk-adapted management of PTC patients based on the response to initial therapy.

## 1. Introduction

The conventional initial therapy for differentiated thyroid cancer (DTC) is surgery, followed by radio-iodine therapy in selected cases [1,2,3,4,5]. In recent years, there has been renewed interest in thyroid cancer management paradigms that use individualized risk assessments as the basis for treatment and follow-up recommendations [6,7,8,9,10,11,12,13,14,15,16,17,18,19,20].

Routinely, the initial treatment (total thyroidectomy and radioiodine treatment after surgery) is performed according to the American Thyroid Association (ATA) risk criteria (low, intermediate and high) that define the risk of persistent/recurrent disease in DTC patients [1]. This initial risk assessment is used to define the time and frequency of follow-up during the first year after surgery [1,21]. Therefore, it is pivotal to identify the patients who are in remission after initial treatment in order to reduce the frequency of their follow-up due to a low risk of recurrence; it is also necessary to use individualized management and appropriate testing in patients that might have persistent or recurrent disease after initial therapy [1,2,6,7,8,9,22].

The individualized management approach is based on the response to therapy after initial treatment [1]. The response to treatment is described by four categories: excellent response (ER), biochemical incomplete response (BIR), structural incomplete response (SIR) and indeterminate response (IR). These categories are based on the post-treatment values of thyroglobulin (Tg) (Immulite 2000 Thyroglobulin; DPC, Los Angeles, CA, USA) and thyroglobulin antibodies (TgAb) (TOS, Tosoh A1S-600II; Tosoh Corp., San Francisco, CA, USA) and on imaging studies performed during follow-up [1]. Tuttle et al. demonstrated, for the first time, that the response to the initial treatment is able to predict the risk of having recurrent or persistent disease in DTC patients treated with total thyroidectomy and radioiodine therapy. The authors showed that patients with an initial excellent response had a better outcome compared to patients with initial biochemical or structural incomplete responses [23]. In another retrospective study by Castagna et al., the percentage of patients who achieved cure at the end of follow-up was 90% in low-risk patients, whereas it was 96% in patients who had an excellent response to initial treatment [24]. More importantly, the percentage of patients with high dynamic risk (i.e., patients with initial biochemical or structural incomplete responses) who were cured decreased from 60% to 27%. The positive predictive value (PPV) of dynamic risk stratification was 72.8%, and the PPV of recurrence risk stratification was 39.2%, which means that dynamic risk assessment has a greater ability to predict the response to treatment.

Many other studies confirmed these results [25,26] and, to date, the long-term follow-up of DTC patients is planned according to the response to the initial treatment [1].

In this study, we assumed that the prognostic value of the response to initial therapy might improve by integrating the ATA risk stratification criteria. To demonstrate our hypothesis, we assessed the long-term outcome of 704 patients with papillary thyroid cancer (PTC) based on the response to the initial therapy (excellent response, indeterminate response, or biochemical or structural incomplete response) and the ATA risk stratification criteria (low-, intermediate- and high-risk).

## 2. Materials and Methods

This retrospective study included low- and intermediate-risk PTC patients followed up for a median time of 8 years at the Endocrine Unit of the University Hospital of Siena, Italy. An informed consent form was signed by each enrolled patient and the study was approved by our ethical committee (Ethics Committee of Region Toscana, Area Vasta Sud Est, AOUS. Protocol ID: 10167). The clinical-pathological features of the patients, the biochemical and morphological data were collected at the time of surgery and during the follow-up. Patients were treated according to 2009 ATA guidelines [22] and patients treated from 2016 to the data lock point were treated according to 2015 ATA guidelines [1]. PTC patients were treated with lobectomy or total thyroidectomy with or without radioiodine therapy. After surgery, patients were classified into a low- or intermediate-risk class according to the ATA risk classification system [1].

According to the 2015 ATA guidelines [1], the patients’ clinical status was defined using the following four categories: excellent response (ER), biochemical incomplete response (BIR), indeterminate response (IR) and structural incomplete response (SIR). ER was defined as stimulated/suppressed thyroglobulin (Tg) < 1 ng/mL or undetectable, negative thyroglobulin antibodies (TgAb) and no functional or structural evidence of disease. BIR was defined as suppressed Tg > 1 ng/mL or stimulated Tg > 10 ng/mL, and rising TgAb, if present, with no structural evidence on imaging. IR was defined as Tg and TgAb still detectable but not rising significantly (<20%), suppressed Tg level 0.2–1 ng/mL, stimulated Tg > 1 but <10 ng/mL, stable thyroid bed lesions and no features of malignancy, neck lymph nodes < 1 cm and stable with no features suggestive of malignancy or persistence and weak/negligible uptake in the neck on a nuclear scan. SIR was defined as local, regional or distant disease with any Tg or TgAb levels. The clinical status of PTC patients was assessed at two different time points: 6–12 months after initial treatment (surgery ± radioiodine therapy; response to initial therapy) and a median follow-up of 8 years (final outcome). Recurrence of disease (RD) was defined as the development of biochemical or structural disease, in patients with previous excellent response, at any time during the follow-up [1].

**Figure 1:**
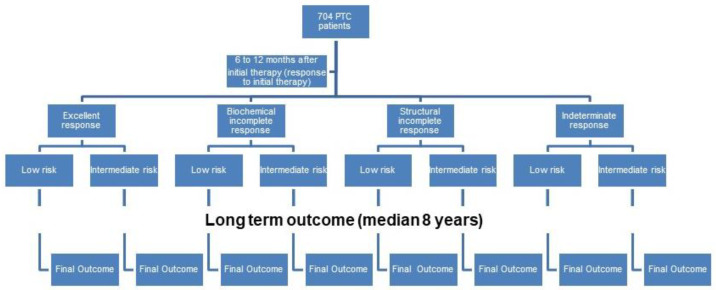
The response to initial therapy and the long-term outcome evaluated for the eight subgroups.

According to the 2015 ATA guidelines [1], in order to evaluate the response to the initial therapy, each subgroup of patients was divided into two subgroups according to the initial ATA risk criteria. Finally, we had 8 subgroups of PTC patients: (1) low-risk PTC with excellent response; (2) intermediate-risk PTC with excellent response; (3) low-risk PTC with indeterminate response; (4) intermediate-risk PTC with indeterminate response; (5) low-risk PTC with biochemical incomplete response; (6) intermediate-risk PTC with biochemical incomplete response; (7) low-risk PTC with structural incomplete response; and (8) intermediate-risk PTC with structural incomplete response. The long-term outcome at a median follow-up of 8 years was evaluated for the eight subgroups (Figure 1).

### Statistical Analysis

Epidemiological data are presented as mean ± SD and median when appropriate. The *t* test for independent data was performed for normal variables. To evaluate significant differences in data frequency, we analyzed contingency tables. Tables with a size larger than 2 × 2 were analyzed using the Chi-squared test or Fisher exact test, when all cell frequencies were or were not greater than four, respectively. Logistic regression was used to estimate the effects of several clinical and pathological features (gender, age at diagnosis, multifocality, bilaterality, minimal extrathyroidal extension and ATA risk class) on the worst long-term clinical outcome (persistent/recurrent structural disease) in PTC patients.

To perform logistic regression analysis, patients were classified into two groups: (1) patients with excellent response if there was no clinical, radiological or cytological/histological evidence of disease and (2) patients with worse clinical outcome if they had persistent/recurrent disease. Receiver operating characteristic (ROC) curves were used to assess the performance of each scoring system. A comparison between the area under the ROC curves (AUC) was performed using DeLong’s test for dependent ROC curves. Statistical analysis was performed using the SPSS Statistics version 22.0. A *p*-value < 0.05 was considered statistically significant.

## 3. Results

### 3.1. Clinical–Pathological and Epidemiological Features of PTC Patients

A total of 704 PTC patients, referred to our center and followed up for a median time of 8 years, were enrolled in the study. Overall, 372/704 (52.8%) were classified as low-risk PTC and 332/704 (47.2%) as intermediate-risk PTC. In the low-risk group, 362/372 (97.3%) patients were treated with total thyroidectomy and 10/372 (2.7%) patients with lobectomy. A total of 267/372 (71.8%) patients were treated with radioiodine therapy after surgery. In the intermediate-risk group, all patients were treated with total thyroidectomy and 325/332 (97.9%) were also treated with radioiodine therapy after surgery. As expected, according to the American Joint Committee on Cancer Union for International Cancer Control Tumor, Node, Metastasis (TNM) staging system [25,27], the intermediate-risk PTC patients more frequently had tumors classified as T3 (69.3% versus 4.3% in low-risk patients, *p* < 0.0001) and lymph node metastases at diagnosis (50.3% versus 0% in low-risk patients, *p* < 0.0001). Also, tumor multifocality (44.6% versus 30.6%, *p* = 0.0001) and bilaterality (35.5% versus 18.0%, *p* < 0.0001) were more common in the intermediate-risk group than in the low-risk patients, respectively. The epidemiological, clinical and pathological features of these 704 patients are reported in Table 1.

### 3.2. First Evaluation after Initial Treatment

At the time of the first follow-up visit, performed 6–12 months after initial treatment, PTC patients were divided into four subgroups according to the response to the initial therapy: excellent response, indeterminate response and biochemical and structural incomplete responses. As expected, the response to initial therapy was significantly different between the low- and intermediate-risk groups (*p* < 0.0001) (Table 2). The rate of excellent response was significantly higher in the low-risk group than in the intermediate-risk group (86% versus 60.8%), while a lower rate of persistent biochemical or structural disease was observed in the low-risk patients (8.3% and 2.7%, respectively) compared to the intermediate-risk group (15.4 and 16.9%, respectively) (Table 2).

### 3.3. Long-Term Outcome in the Whole Group According to the Initial ATA Risk Class

**Figure 2:**
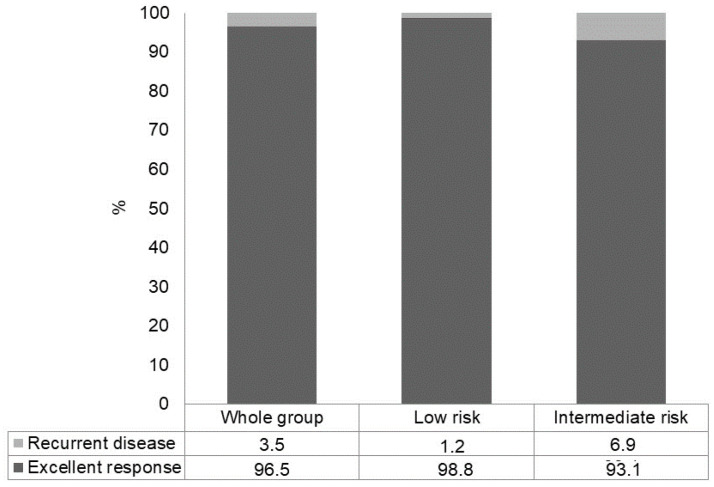
Recurrent disease at a median follow-up of 8 years in patients with excellent response after initial therapy, in the whole group and according to the initial ATA risk class.

After a median follow-up of 8 years, in the initial excellent response subgroup of PTC patients (low- plus intermediate-risk patients) (*n* = 522), a persistent excellent response was observed in 504/522 (96.5%) patients, whereas recurrent disease was found in 18/522 (3.5%) patients (Figure 2). Nevertheless, the rate of recurrent disease was significantly higher in intermediate-risk PTC patients compared to low-risk PTC patients (6.9% versus 1.2%, *p* = 0.0005) (Figure 2).

**Figure 3:**
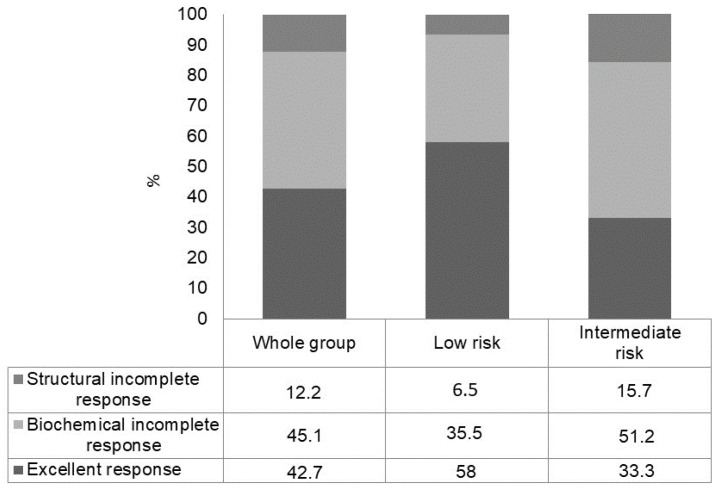
Outcome at a median follow-up of 8 years in patients with biochemical incomplete response after initial therapy, in the whole group and according to ATA risk class.

In the initial biochemical incomplete response subgroup (low- plus intermediate-risk patients) (n = 82), in the long-term follow-up, an excellent response was observed in 35/82 (42.7%) patients, a persistent biochemical incomplete response in 37/82 (45.1%) patients and a structural incomplete response in 10/82 (12.2%) cases (Figure 2). The rate of excellent response was significantly higher in low-risk PTC patients (58.0%) than in intermediate-risk PTC patients (33.3%) (*p* = 0.007) (Figure 3).

**Figure 4:**
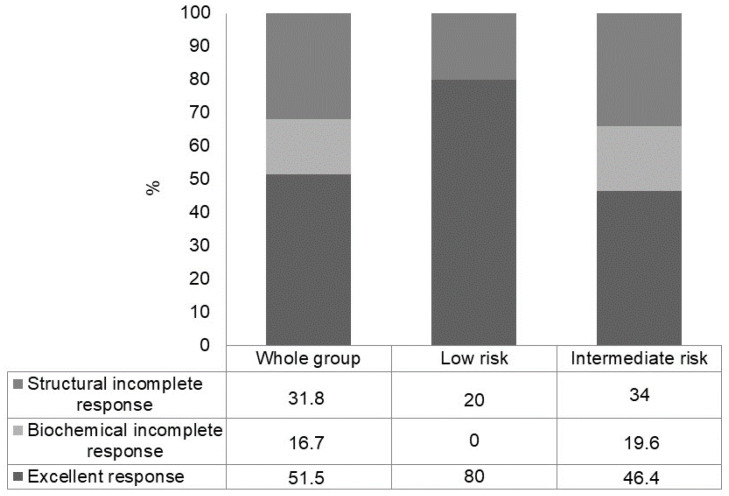
Outcome at a median follow-up of 8 years in patients with structural incomplete response after initial therapy, in the whole group and according to ATA risk class.

In the initial structural incomplete response subgroup (low- plus intermediate-risk patients) (n = 66), after additional treatments, an excellent response was observed in 34/66 (51.5%) patients, a biochemical incomplete response in 11/66 (16.7%) patients and a structural incomplete response in 21/66 (31.8%) patients (Figure 3). Also in this case, the rate of excellent response was higher in low-risk patients (80.0%) than in intermediate-risk patients (46.4%) (*p* = 0.08) (Figure 4).

Moreover, all patients with an initial indeterminate response had an excellent response at the last follow-up visit, regardless of the initial ATA risk class.

### 3.4. Predictors of Long-Term Outcome

We also evaluated possible predictors of outcome in each subgroup of patients: excellent response, biochemical incomplete response and structural incomplete response. In patients with an excellent response after initial therapy, extrathyroidal tumor extension (*p* = 0.01) and ATA intermediate-risk class (*p* = 0.002) correlated with a higher risk of recurrence during follow-up (Table 3). The correlation between outcome and ATA intermediate-risk class was confirmed in the multivariate analysis (*p* = 0.002).

In patients with a biochemical incomplete response after initial therapy, male gender (*p* = 0.04) and ATA intermediate-risk class (*p* = 0.03) correlated with a higher risk of persistent disease (biochemical or structural) during follow-up (Table 3). The correlation of the outcome with male gender and ATA intermediate-risk class was confirmed in the multivariate analysis (*p* = 0.03 and *p* = 0.02, respectively).

In patients with a structural incomplete response after initial therapy, extrathyroidal tumor extension (*p* = 0.04) correlated with a higher risk of persistent disease (biochemical or structural) during follow-up, while ATA intermediate-risk class was close to statistical significance (*p* = 0.06) (Table 3). In the multivariate analysis, older age at diagnosis (*p* = 0.04) and ATA intermediate-risk class (*p* = 0.03) were independently associated with a higher risk of persistent disease (Table 3).

### 3.5. Prognostic Performance of ATA Risk Class, Response to Initial Therapy and ATA Risk Classes Plus Response to Initial Therapy in PTC Patients

**Figure 5:**
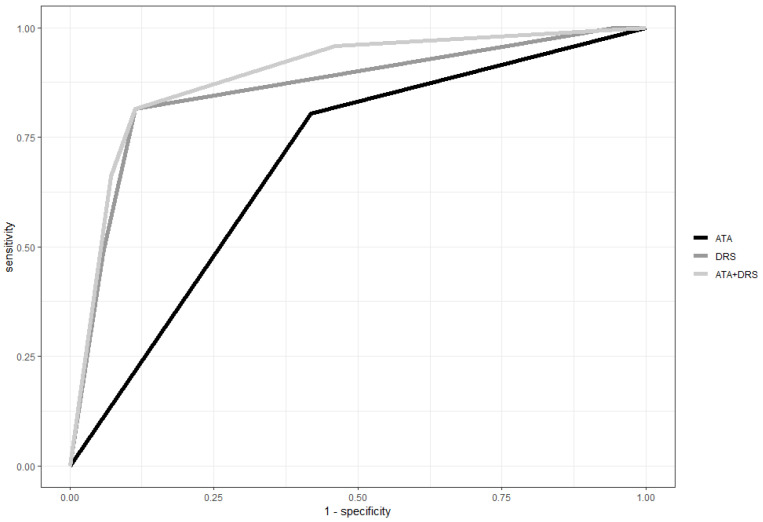
ROC curve analysis: the accuracy in predicting the long-term outcome is significantly improved after integrating ATA risk classes with the response to initial therapy.

To assess the overall prognostic performance of the ATA risk categories of response to initial therapy and that of the combination of ATA risk class and response to initial therapy, an ROC curve analysis was performed. The overall prognostic performance, defined by the ROC curve analysis, of response to initial therapy integrated with the ATA risk system (AUC: 0.89; 95% CI: 0.86–0.92) was significantly higher compared to the ATA risk stratification (AUC 0,69; 95% CI: 0.65–0.74, *p* < 0.001) and DRS systems alone (AUC: 0.86 95% CI: 0.82–0.90, *p* = 0.007) (Figure 5).

## 4. Discussion

The management of differentiated thyroid cancer (DTC) patients should be based on a risk-adapted approach to optimize individual patient care [1]. The initial treatment (extension of surgery and/or radioiodine therapy) depends on the individualized risk of recurrence and the risk of disease-specific mortality according to the ATA risk criteria and the AJCC/TNM staging system [1,22,25].

The subsequent follow-up of DTC patients is based on the response to the initial treatment, which may be defined as an excellent response, indeterminate response, biochemical incomplete response or structural incomplete response [1].

The ongoing dynamic evaluation of response to treatment is used to monitor DTC patients during long-term follow-ups. This strategy allows for customized care by providing an ongoing re-evaluation of a patient’s clinical status and follow-up plan based on the data collected during the follow-up [23]. Patients with an excellent response can be referred for a less intensive follow-up (without a difference between low- and intermediate-risk class PTC patients), while a more intensive follow-up is indicated in patients with persistent biochemical or structural incomplete responses.

Based on previous studies [23,26], we observed that the rate of excellent response, at the last follow-up visit, decreased progressively starting from patients with an initial excellent response to patients with a structural incomplete response after initial therapy (96.5% in the excellent response group to 51.5% in the structural incomplete response group). These results confirm the better ability of the response to initial therapy to predict the long-term outcome when compared to the predictive value of the initial risk stratification system proposed by ATA and the European Thyroid Association (ETA) [1,3]. We also observed that all patients with an initial indeterminate response had an excellent response at the last follow-up visit. However, we assumed that the long-term outcome may be also influenced by the initial ATA risk class (low-, intermediate- or high-risk) [1].

To verify our hypothesis, we evaluated the disease status at long-term follow-up in eight different subgroups of PTC patients, defined according to the response to initial therapy (excellent response, indeterminate response or biochemical or structural incomplete responses) and the ATA risk classes (low-, intermediate- or high-risk) [1].

We found that the initial ATA risk class significantly affected the long-term outcome in all but one category of response to the initial treatment. The rate of clinical remission at the last follow-up visit was higher in low-risk patients compared to intermediate-risk patients. In particular, the percentage of remission was 98.8% and 93.1% in ATA low- and intermediate-risk patients, respectively (*p* = 0.0005), in patients with an excellent response after initial therapy; 58.0% and 33.0% in ATA low- and intermediate-risk patients, respectively (*p* = 0.007), in patients with a biochemical incomplete response; and 80.0% and 46.4% in ATA low- and intermediate-risk patients, respectively (*p* = 0.08), in patients with a structural incomplete response. Likewise, the rate of recurrent disease after a period of excellent response was significantly higher in the intermediate-risk PTC patients compared to the low-risk PTC patients (6.9% versus 1.2%, *p* = 0.0005).

To date, only a few studies have assessed the prognostic role of the ATA risk criteria integrated with the response to initial therapy [28,29,30]. Seejore et al. evaluated a large cohort of 756 DTC patients who achieved an excellent response to treatment at the first evaluation after initial therapy. Only 15 patients (2.0%) developed radiological disease recurrence after a median follow-up of 11.2 years. They further analyzed the outcomes according to postoperative ATA risk stratification and showed that patients with an excellent response after initial therapy and classified as having a high-risk disease according to the ATA guidelines had an almost threefold higher recurrence rate (2/34 (5.9%) vs. 13/722 (1.8%)) than those with ATA low- or intermediate-risk disease [28].

Moreover, a retrospective study of 83 patients with a biochemical incomplete response after initial therapy was performed to evaluate factors associated with long-term clinical outcome and predictors of structural recurrence in these patients [29]. During a mean follow-up of 12 ± 6.6 years, 49 (59%) patients remained with a BIR or switched to no evidence of disease, while 34 (41%) progressed to structural disease. At the last follow-up visit, a disease-related death was recorded in three cases (3.6%). In this study, the rates of cure in patients with a biochemical incomplete response were 85.1%, 10.6% and 4.3% in the low-, intermediate- and high-risk classes, respectively (*p* < 0.001).

The American Thyroid Association (ATA) initial risk stratification system and/or the AJCC/TNM (8th ed.) staging system at diagnosis predicted the shift from BIR to structural disease, regardless of patients’ postoperative Tg levels.

It is important to underline that, in both of these studies, the prognostic role of the combination of ATA risk criteria and response to initial therapy was evaluated only in univariate analyses. Conversely, we demonstrated that the ATA risk criteria are independently associated with the long-term follow-up in all patients dynamically risk-stratified in the multivariate analysis. Moreover, in these studies [28,29], only selected subgroups of patients were evaluated (excellent response or biochemical incomplete response), while, in our study, we assessed all subgroups of response to initial therapy in a series of PTC patients followed at the same institution. Finally, we evaluated, for the first time, the prognostic value of two different stratification systems alone and in combination using an ROC curve analysis. The prognostic value of response to initial therapy integrated with the ATA risk system (AUC: 0.89; 95% CI: 0.86–0.92) was higher when compared to ATA risk criteria and response to initial therapy alone, suggesting that this approach is able to better predict the long-term outcome of PTC patients at a low and intermediate risk of developing a persistent/recurrent disease.

Our study has some limitations: It was retrospective and included patients treated when the management of DTC was based on the “one size fits all” approach. Moreover, the prognostic role of integrating the ATA risk stratification systems and response to initial therapy was not evaluated in ATA high-risk PTC patients due to the small number of these patients in our cohort. On the other hand, the strengths of our study were the large cohort of patients, the long-term follow-up (median: 8 years), a consecutive enrollment of the patients and the same management approach for the disease, which minimized potential differences due to various treatment approaches.

## 5. Conclusions

In summary, this study of a large cohort of low- and intermediate-risk PTC patients showed a higher rate of cure in low-risk patients compared to intermediate-risk patients, showing that low-risk PTC patients have a better long-term outcome than intermediate-risk patients in each subgroup of patients dynamically stratified after initial therapy. In order to customize the management of these patients to detect a clinically significant disease but reduce the frequency of exams during the follow-up, it is pivotal to know all the possible predictive factors of the worst clinical outcome. This study confirms that the response to initial therapy is predictive of the long-term outcome in PTC patients, but it also underlines that the initial ATA risk class may be useful for improving the risk-adapted management of PTC patients.

## Figures and Tables

**Table 1:** Epidemiological and clinical–pathological features of PTC patients.

	Total Patients (*n* = 704)	Low-Risk Patients (*n* = 372)	Intermediate-Risk Patients (*n* = 332)	*p*-Value
**Sex, n (%)**				0.3
**M**	183 (26.0%)	103 (27.7%)	80 (24.1%)	
**F**	521 (74.0%)	269 (72.3%)	252 (75.9%)	
**Age at diagnosis, yrs**				0.1
**Mean**	46.2	47.6	44.8	
**Median**	45.0	48.0	43.0	
**Surgical treatment, n (%)**				<0.05
**Total thyroidectomy**	694 (98.6%)	362 (97.3%)	332 (100%)	
**Lobectomy**	10 (1.4%)	10 (2.7%)	0 (0%)	
**Histological variant**				<0.0001
**Classical**	18/273 * (6.6%)	8/142 (5.6%)	10/131 (7.7%)	
**Follicular**	171/273 * (62.6%)	134/142 (94.4%)	37/131 (28.2%)	
**Aggressive variant (hurtle cells, tall cells, solid, Warthin-like, columnar cells, trabecular, insular, sclerosant)**	84/273 * (30.8%)	0/142 (0%)	84/131 (64.1%)	
**T stage, n (%)**				<0.0001
**1**	375 (53.3%)	290 (78.0%)	85 (25.6%)	
**2**	83 (11.8%)	66 (17.7%)	17 (5.1%)	
**3**	246 (34.9%)	16 (4.3%)	230 (69.3%)	
**Multifocal, n (%)**	262 (37.2%)	114 (30.6%)	148 (44.6%)	0.0001
**Bilateral, n (%)**	185 (26.3%)	67 (18.0%)	118 (35.5%)	<0.0001
**Extrathyroidal, n (%)**	225 (31.9%)	0 (0%)	225 (67.8%)	<0.0001
**Lymph node metastases, n (%)**	167 (23.6%)	0 (0%)	167 (50.3%)	<0.0001
**TNM Stage, n (%)**				<0.0001
**I**	622 (88.4%)	366 (98.4%)	256 (77.1%)	
**II**	82 (11.6%)	6 (1.6%)	76 (22.9%)	
**Post-op I131 treatment, n (%)**	592 (84.1%)	267 (71.8%)	325 (97.9%)	<0.0001
**Follow-up**				0.06
**Mean**	8.3 ± 5.0	8.6 ± 5.4	8.0 ± 4.7	
**Median**	8.0	8.0	7.6	
**Range**	1–54	1–54	1–28.7	

* Data are available from only 273/704 patients.

**Table 2:** Dynamic risk stratification in PTC patients at first evaluation after initial treatment.

	Excellent Response	Indeterminate Response	Biochemical Incomplete Response	Structural Incomplete Response
**Total patients (*n* = 704)**	522 (74.2%)	34 (4.8%)	82 (11.6%)	66 (9.4%)
**Low-risk patients (*n* = 372)**	320 (86.0%)	11 (3.0%)	31 (8.3%)	10 (2.7%)
**Intermediate-risk patients (*n* = 332)**	202 (60.8%)	23 (6.9%)	51 (15.4%)	56 (16.9%)
**Low- vs. intermediate-risk patients (*p*-value)**	<0.0001			

**Table 3:** Univariate and multivariate analysis for recurrent or persistent (structural or biochemical) disease in the 3 subgroups of patients defined according to the response to initial treatment.

	Univariate Analysyis				Multivariate Analysis		
Response to Initial Treatment	Parameters	OR	95%CI	*p* Value	OR	95%CI	*p* Value
**Excellent response**	Age >55 years	0.61	0.17–1.74	0.39	-	-	-
	Male gender	1.61	0.55–4.26	0.34	-	-	-
	Multifocality	2.39	0.92–6.36	0.07	-	-	-
	Bilaterality	2.15	0.77–5.59	0.12	-	-	-
	**mETE**	**3.48**	**1.34–9.31**	**0.01**	-	-	-
	**Intermediate ATA risk class**	**5.88**	**2.07–20.98**	**0.002**	**5.88**	**2.07–20.98**	**0.002**
**Biochemical incomplete response**	Age >55 years	1.43	0.53–4.05	0.48	-	–	-
	**Male gender**	**2.72**	**1.05–7.56**	**0.04**	**2.92**	**1.04–8.48**	**0.03**
	Multifocality	1.01	0.41–2.50	0.96	-	–	-
	Bilaterality	1.12	0.44–2.91	0.80	-	–	-
	mETE	1.48	0.59–3.79	0.40	-	–	-
	**Intermediate ATA risk class**	**2.76**	**1.11–7.09**	**0.03**	**2.95**	**1.15–7.91**	**0.02**
**Structural incomplete response**	Age >55 years	2.63	081–9.50	0.11	**4.09**	**1.09–20.0**	**0.04**
	Male gender	0.96	0.34–2.65	0.93	-	–	-
	Multifocality	1.42	0.54–3.82	0.47	-	–	-
	Bilaterality	0.84	0.30–2.31	0.74	-	–	-
	**mETE**	**2.78**	**1.03–7.86**	**0.04**	-	–	-
	**Intermediate ATA risk class**	**4.61**	**1.04–32.43**	**0.06**	**7.32**	**1.43–62.2**	**0.03**

Legend of the table: mETE (minimal extrathyroidal extension); ATA (American Thyroid Association); OR (odds ratio); 95% CI (95% confidence interval).

## Data Availability

The data presented in this study are available on request from the corresponding author. The data are not publicly available due to patients’ privacy.
